# Dynamic in situ growth of bonded-phase silica nanospheres on silica capillary inner walls for open-tubular liquid chromatography

**DOI:** 10.1007/s00216-023-04798-1

**Published:** 2023-06-23

**Authors:** Mohamed Adel Ahmed, Alireza Ghiasvand, Joselito P. Quirino

**Affiliations:** 1 Australian Centre for Research On Separation Science (ACROSS), School of Natural Sciences, University of Tasmania, Hobart, TAS 7001 Australia; 2 Department of Analytical Chemistry, Lorestan University, Khoramabad, Iran

**Keywords:** Silica nanospheres, Dynamic in situ synthesis, Open-tubular liquid chromatography, Open-tubular capillary electrochromatography

## Abstract

Silica nanospheres (SNS) were grown on the inner walls of silica capillaries through a dynamic in situ nucleation process to prepare a highly porous and large accessible surface area substrate. The SNS were then functionalized with octadecyl (C_18_), 3-aminopropyltriethoxysilane (APTES), beta-cyclodextrin (*β*-CD), and amino groups to develop robust and efficient chromatographic stationary phases. The modified silica capillaries were exploited for open-tubular liquid chromatography (OT-LC) and open-tubular capillary electrochromatography (OT-CEC) applications. The prepared stationary phases were compared to conventional capillaries in terms of separation performance. The synthesis process was optimized, and the bonded-phase stationary phases were characterized by the electron microscopy technique. The effects of different solvents, additives, and functional groups on the geometry and chromatographic resolving power of the SNS were envisaged. The capillaries modified with octadecyl groups were evaluated for the separation of non-steroidal anti-inflammatory drugs, phenones, alkenylbenzenes, and enantiomers of chlorophenoxy herbicides. As an application instance, an SNS-C_18_-coated capillary was utilized for the separation of alkenylbenzenes from clove extract and protein digest medium, through OT-LC and OT-CEC techniques, respectively. The *β*-CD functionalized capillary was applied for the OT-CEC separation of a dichlorprop racemic mixture.

**Graphical abstract:**

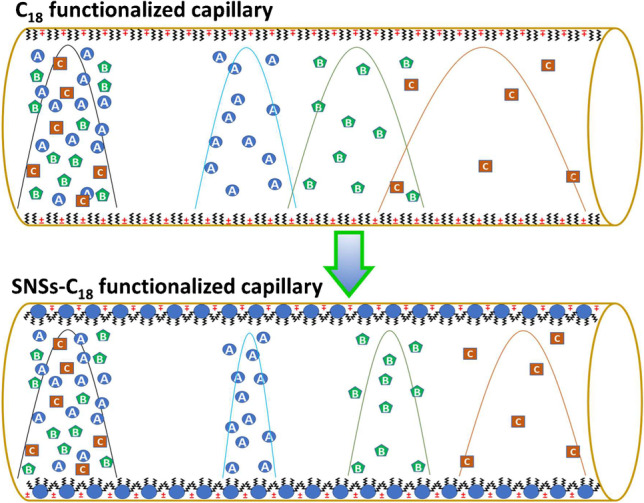

**Supplementary information:**

The online version contains supplementary material available at 10.1007/s00216-023-04798-1.

## Introduction

Silica is a ubiquitous and low-cost natural compound that plays a key role in the analytical separation processes, particularly in the form of fused silica in chromatography and electrophoresis, due to its good sorption features [1]. Nevertheless, the natural specifications of fused silica don’t meet the requirements for the development of miniaturized/portable chromatographic and electrophoretic instruments, while these emerging technologies are anticipated to conquer the future of chromatographic techniques [2]. The main challenge in the development of compact open-tubular liquid chromatography (OT-LC) and open-tubular capillary electrochromatography (OT-CEC) systems is the preparation of short microbore capillary columns with higher resolving power and greater durability [3]. The efforts devoted to addressing this issue have opened a new avenue for innovation in the sorbent materials and chromatographic stationary phases. In open-tubular columns, the stationary phase material is attached to the inner walls of the capillary either chemically or physically, and the background solution/mobile phase is delivered through the column by pressure, in OT-LC, or by electroosmotic flow (EOF), in OT-CEC. Open-tubular capillary separations offered great advantages over the classical LC and CEC modes with packed columns. They can be utilized without complicated and expensive instrumentation, offering more robust separations and remarkable resource and time savings [4]. They enable the development of high-performance miniaturized instruments, by surmounting high solvent consumption, band broadening, and high backpressure, corresponding to the stationary phase particles, column frits, and connections in packed columns [5].

However, conventional open-tubular capillaries suffer from disadvantages like low sorption surface area and insufficient stability of the stationary phases which reflect on the low phase ratio and short lifespan of the capillaries, respectively [6, 7]. Different new measures have been presented to increase the phase ratio and durability of the stationary phases by etching [8], and coating the inner walls of the capillaries using polymerized monolith- [9–11] or nanoparticle-based porous layers [12–14]. One of the successful approaches to preparing durable and high surface area stationary phases was the utilization of silica nanospheres (SNS) because they offer a high phase ratio and remarkable physicochemical stability. New procedures provide controlled preparation of SNS with different particle sizes and desired morphologies, to improve their sorption efficiency and applications in separation science, biomedical uses, drug delivery, bio-imaging, and catalyst [15]. Synthesis of SNS through sol–gel chemistry was originally introduced by Stöber et al. [16] in 1968 and since then it had been the backbone of many new routes for the preparation of stationary phases for chromatographic and analytical separation purposes. The surface of SNS can be further modified with different coating materials during or after the formation of the particles to extend their applications and improve their selectivity for the separation of multiple types of analytes [17–19].

The approach of the preparation of porous silica layers in fused silica capillaries for OT-LC was first introduced by Tock et al. [20, 21]. Many efforts were done since then to increase the loadability, stability, and surface area of the stationary phases for more retention of analytes such as polymer-prepared coatings [22–24]. Collins et al. [25] introduced a monolithic polymerized porous layer open-tubular (PLOT) column by polymerization of polystyrene-divinylbenzene (PS-DVB) and butyl methacrylate-ethylene dimethacrylate (BMA-EDMA) by thermal initiation on trimethoxysilylpropyl methacrylate (TMSPMA), which allowed controlling the thickness and morphology of the stationary phase. Liu et al. [26] prepared a cage-like silica nanoparticle-functionalized stationary phase for high-performance capillary electrochromatography (HPCEC) through a one-pot synthesis procedure. This stationary phase inherited hydrophobic and cation-exchange interaction properties. Guo and Colon [27–29] reported a series of articles on the preparation of PLOT capillary columns for OT-LC and OT-CEC separations via sol–gel process to enhance both the retention and stability in alkali and acidic mobile phase/background solutions. In another innovative series, Forster et al. [30–32] reported the synthesis of silica-based thick-film monolithic PLOT capillary columns using a sol–gel process for normal-phase LC (without surface modification) and reversed-phase LC (after surface modification with C_8_). Freitag and Constantin [33] discussed the factors that control the performance of open-tubular capillary columns bearing silica-based C_8_ stationary phases during the sol–gel preparation. They evaluated the resolution and capacity factor of the prepared capillaries for OT-CEC analysis of polycyclic aromatic hydrocarbons (PAHs). In a different study, Gong et al. [34] reported the application of amino-functionalized silica nanoparticles in the background solution as an additive to improve the enantioselective resolution of alkaline drugs by CE. In the abovementioned studies, the solid support (i.e., fused silica) has been functionalized without significant change in its morphological specifications. Therefore, any improvement in the physicochemical properties of the sorbent depends only on the coated or bonded nanoparticles themselves.

In addition to all the above, the solid support can also be modified to provide more desirable physiochemical features like higher accessible surface area and stability for subsequent functionalization. Accordingly, in this research, the inner walls of silica capillaries were coated with SNS using a dynamic in-situ sol–gel method to obtain a large surface area and highly porous substrate. In the second stage, the surface of SNS was functionalized with different chemical groups to prepare the desirable chromatographic stationary phases. The sol–gel process was conducted with different solvents in the presence of neutral, cationic, and anionic surfactants and their effects on the synthesis conditions, particle size, and chromatographic performance of SNS were evaluated. The surface of the SNS formed on the capillary inner wall was chemically modified by beta-cyclodextrin (*β*-CD) and the stationary phase was utilized for OT-LC chiral separation. The SNS-coated capillary, functionalized by hydrophobic C_18_ groups under different synthesis conditions, was employed for the OT-LC separation of alkenylbenzenes in clove extract. It was also evaluated for OT-CEC analysis of tryptic digest of bovine serum albumin (BSA) and the results were compared with a PDDA-PSS (polydiallyldimethylammonium chloride-poly sodium styrene sulfonate) capillary.

## Experimental

### Materials and solutions

Tetraethyl orthosilicate (TEOS, 98%), (3-aminopropyl) triethoxysilane (APTES, 99%), and octadecyltrichlorosilane (OTS, ≥ 90%) were purchased from Sigma-Aldrich (NSW, Australia). Acetone, ethanol (EtOH), methanol (MeOH), acetonitrile (ACN), carbonyldiimidazole, chloroform (CHCl_3_), cyclohexane, toluene, beta-cyclodextrin (*β*-CD), cetyltrimethylammonium bromide (CTAB), dimethylformamide (DMF), sodium dodecyl sulfate (SDS), polyvinylpyrrolidone (PVP), n-propylamine, ammonium bicarbonate (NH_4_HCO_3_), ammonium hydroxide solution (28–30% NH_3_), formic acid (FA, ≥ 95%), n-propylamine, TRIS hydrochloride (Tris–HCl), DL-dithiothreitol (DDT), polystyrene sulfonate (PSS), poly(diallyldimethylammonium chloride) (PDDAC), trypsin, bovine serum albumin (BSA, ≥ 98%), and indole-3-acetic acid sodium salt (IAA, ≥ 98%) were obtained from Sigma-Aldrich. Unless otherwise stated, all reagents used were at least of analytical reagent grade. Ultrapure Milli-Q water (MQW) was supplied by a Milli-Q purification system (Millipore, Bedford, MA, USA). Stock standard solutions (2000 µg mL^−1^) of phenones (butyrophenone, valerophenone, and hexanophenone), non-steroidal anti-inflammatory drugs (NSAIDs) including ketoprofen, ibuprofen, and fenbufen, and racemic analytes (dichlorprop) were prepared in methanol.

### Instrumentation

All OT-LC and OT-CEC experiments were conducted using an Agilent HP G1600AX 3D CE (Agilent Technologies, Santa Clara, USA), equipped with a UV detector at 200 nm and operated by 3D-CE ChemStation (Rev.B.03.01.317) software. Uncoated fused silica capillaries (50, 75, 100, and 150 μm I.D.) were provided by Polymicro Technologies (Phoenix, AZ, USA). The scanning electron microscope (SEM) images of the capillaries’ cross sections were recorded using a Hitachi SU-70 scanning electron microscope (Chiyoda, Tokyo, Japan).

### Pressurized chamber for dynamic coating of silica capillaries

**Fig. 1:**
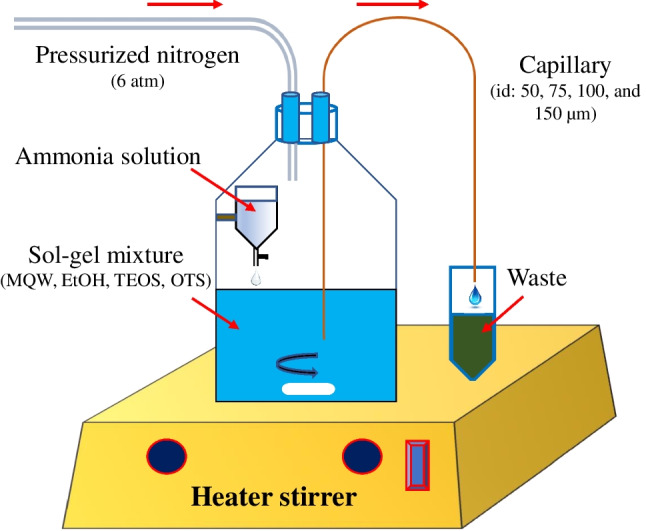
Schematic representation of the pressurized chamber for dynamic coating of capillaries

A home-built pressurized coating chamber was fabricated. For this purpose, the blue screw cap of a 250 mL DURAN® pressure plus borosilicate reagent bottle was replaced by an HPLC mobile phase container’s cap, equipped with airtight flangeless fittings. One of the cap’s distributors was connected to the laboratory pressurized nitrogen pipeline and the other distributor was connected to a fused silica capillary. The tip of the capillary was submerged in the sol–gel medium inside the chamber as can be seen schematically in Fig. 1. The chamber could withstand pressures up to 6 atm safely. For the simultaneous coating of several capillaries with the same conditions, a container’s cap with more than two distributors can be used and for the simultaneous coating of several capillaries at different coating conditions, several chambers with various reaction contents can be serially coupled together using one pressurized nitrogen tube, as shown in Supplementary Material, Fig. S1. The capillaries were cleaned by flushing with NaOH solution (1 M) for 30 min, MQW for 10 min, and MeOH for 15 min, followed by purging with pressurized nitrogen for 10 min, before starting the coating process.

### In situ synthesis of SNS on the inner walls of fused silica capillary

The SNS were synthesized on the inner surface of fused silica capillaries through hydrolysis and condensation of TEOS in ethanol, in the presence of ammonia as the morphological catalyst. First, 6.5 mL of TEOS and 21 mL of ethanol were transferred into the coating chamber and sonicated for 10 min in ambient conditions. Then, the chamber was sealed and pressurized, to pump the TEOS solution through the capillary. A special container was designed and implemented inside the chamber for dropwise delivery of ammonia to the sol–gel solution. It was loaded with 5.6 mL 0.5 M ammonia solution to be added to the synthesis solution. After 3 h, the chamber was depressurized, the capillary removed, and flushed with ethanol (5 min) in another pressurized chamber to remove the unreacted TEOS. Finally, the capillary was flushed with nitrogen (10 min) and dried in an oven at 95 °C overnight.

### Functionalization of the SNS-coated silica capillary with C_18_

For functionalization of the SNS-coated silica capillary with C_18_, it was washed successively with toluene and nitrogen each for 5 min. A silanization mixture containing 1% (v/v) OTS and 1% (v/v) TEOS in toluene was prepared and passed through the capillary for 1 h for chemical bonding of C_18_ groups to the surface of SNS on the inner wall of the capillary. Unreacted OTS and TEOS residues were washed by flushing the capillary with toluene for 5 min. The capillary was flushed with nitrogen (10 min) and then dried overnight in an oven at 95 °C. For functionalization of the SNS-coated capillary with the amine group, the column was flushed with a 0.33% (v/v) APTES solution in toluene for 5 h. The derivatized column was then sealed from both sides and kept in the oven at 95 °C for 24 h. Finally, the column was washed with toluene for 10 min and dried with nitrogen flow.

### Preparation of SNS-coated fused silica capillaries modified with *β*-CD

The surface of the SNS was first modified with amino groups, to provide a proper reactive spacer group, as demonstrated by Jadhav et al. [35]. For this purpose, an SNS-coated fused silica capillary column was flushed with a mixture of APTES (250 µL) and n-propylamine (2 µL) in cyclohexane (10 mL) for 15 min and then it was sealed at both ends using GC septa and kept in an oven at 60 °C for 1 h. Afterwards, the column was washed with acetone to remove nongrafted APTEPS and other impurities. For chemical bonding of *β*-CD to the amino-terminated groups of the SNS surface, the capillary was filled with a solution of 20 mg *β*-CD and 8 mg carbonyldiimidazole in 0.5 mL dry DMF. The capillary was then sealed at both ends and kept at 60 °C in an oven for 3 h. Finally, the column was dried with nitrogen and flushed with the corresponding BGS.

### Sample preparation

The sample solutions of phenones were freshly prepared from their stock solutions and diluted to appropriate concentrations with the corresponding mobile phase (0.2 mM CTAB in 25 mM phosphate buffer with pH 7.0). The solutions were stored at 4 °C when not in use. The clove whole samples were purchased from a local supermarket (Hobart, Australia). The extract was prepared by sonicating 0.2 g of the clove whole in 10 mL methanol for 15 min followed by 10 min centrifugation at 2000 rpm to remove solid particles. Then, 50-µL portions of the supernatant were dissolved in 350 µL of the mobile phase (100 mM phosphate buffer pH 5.8) and subjected to OT-LC analysis.

The BSA digestion steps were adopted from the literature [36]. Briefly, 0.5 mg BSA was dissolved in a mixture of 50 µL of Tris–HCl (500 mM), 50 μL DDT (50 mM), and 150 μL MQW. The solution was kept at 37 °C for 5 min in a water bath and then 50 μL of IAA (100 μM) was added. The resulting solution was kept in a dark place for 60 min at room temperature followed by dilution with 300 µL of 10 mM ammonium bicarbonate. Afterwards, 50 µL of 0.5 mg mL^−1^ trypsin solution (in 1 mM HCl) was added and the solution was incubated at 37 °C for 16 h. Finally, the reaction was quenched by adding 50 µL of 25% acetic acid. The solution was then diluted in ACN (volume ratio of 1:1) and centrifuged at 2000 rpm for 2 min. The supernatant was sonicated for 20 s before injection into the CE instrument.

### OT-LC and OT-CEC conditions

All OT-LC and OT-CEC experiments on the functionalized capillaries were conducted using the Agilent CE system with the corresponding mobile phase/background solution (BGS). The prepared capillary was first flushed with MQW (1 min) and mobile phase/BGS (2 min) at 1 bar using the internal pressure of the CE instrument. The OT-LC and OT-CEC separations were performed at 50 mbar and ± 10 kV, respectively, unless otherwise stated. Typical injections of standard samples were performed at 25 mbar for 5 s unless otherwise stated. The clove extract was analyzed using OT-LC using a mobile phase of 100 mM phosphate buffer (pH 5.8). The OT-CEC experiments for the BSA digest sample were carried out in a BGS of 0.5 M FA in 20% ACN.

## Results and discussion

### Optimization of the dynamic in situ synthesis procedure

Silica capillaries coated with nanoparticles are nowadays the object of intense research due to their promising features and applications, in different analytical chemistry areas, particularly open-tubular chromatography. These capillaries can be prepared using static, dynamic, sol–gel, and liquid-phase deposition methods. However, the thickness, stability, and uniformity of the coated layer obtained by these strategies are not satisfactory [23].

Most of the reported methods for coating silica capillaries using the sol–gel method are based on the static method, in which the capillary is filled with the reaction medium and allows the reaction to proceed for a long time [22, 23]. The static coating method was first developed by Golay [37] for the preparation of polymeric stationary phases, in which the column is completely filled with a dilute solution that evaporates, leaving a film of stationary phase. This method guarantees a more uniform film. The value of the phase ratio and the thickness of the liquid layer can be easily determined from the volume and stationary phase solution. However, in practice, the dynamic method is used almost exclusively, mainly because it requires relatively simpler equipment. One of the disadvantages of the static method is that when there is a gas bubble or it is created during the evaporation of the solvent in the capillary, this technique fails. Therefore, to minimize the risk of gas bubbles, serious precautions need to be taken. On the other hand, due to the very small volume of the capillary and the small amount of reagents, the coating layer is very thin, and therefore to obtain the appropriate thickness, the coating process must be repeated several times [37, 38]. In the dynamic method, a plug of concentrated solution passes through a capillary column. The dynamic method was first used by Dijkstra and De Goey [39] and examined in detail by Kaiser [40, 41]. It was further improved by Blomberg [42, 43]. The simplicity of this method and the lack of complex equipment make it the most commonly used method [44].

In this study, a combination of dynamic and sol–gel methods was used to coat the fused silica capillary columns with functionalized SNS. SNS-coated capillaries were prepared based on a modified Stöber technique which includes ammonia as a catalyst in the presence of TEOS, as a source of silica in alcoholic media [16].

Before the study of the chemical variables, it was necessary to practically develop a reliable setup for circulating the reaction mixture through the capillary with sufficient pressure and at a constant flow rate to have a uniform and durable coating. The delivery of the coating solution is very integral to the coating uniformity. For this purpose, different pumping methods were tested including the use of dual-piston HPLC pump and peristaltic pump, but they failed. SNS was deposited on the pistons and tubing of the HPLC pump and blocked, while the peristaltic pump did not generate enough pressure for the desired flow rates. Additionally, the SNS formed on the connecting tubes between the pumps and capillaries. Accordingly, a home-built pressurized coating chamber was fabricated by adopting the method reported by Hayes and Malik [45] in 1997. This was the most effective delivery system since it provided enough pressure with a constant flow rate to deliver sol–gel solution directly through the capillary, without any tubing and fittings. However, the viscosity of the sol solution was a still critical parameter and should be controlled to prevent clogging.

In the second stage, the effective chemical variables were evaluated. First, different ratios of TEOS, ethanol, water, and ammonia solution were examined to reach the optimum recipe of the coating solution under ambient conditions. The results revealed that the concentration of ammonia solution was very critical to the morphological features of SNS, while the condensation rate was strongly dependent on the volume of water. High concentrations of ammonia resulted in the quick formation of big-size SNS, which often led to column blockage.

**Fig. 2:**
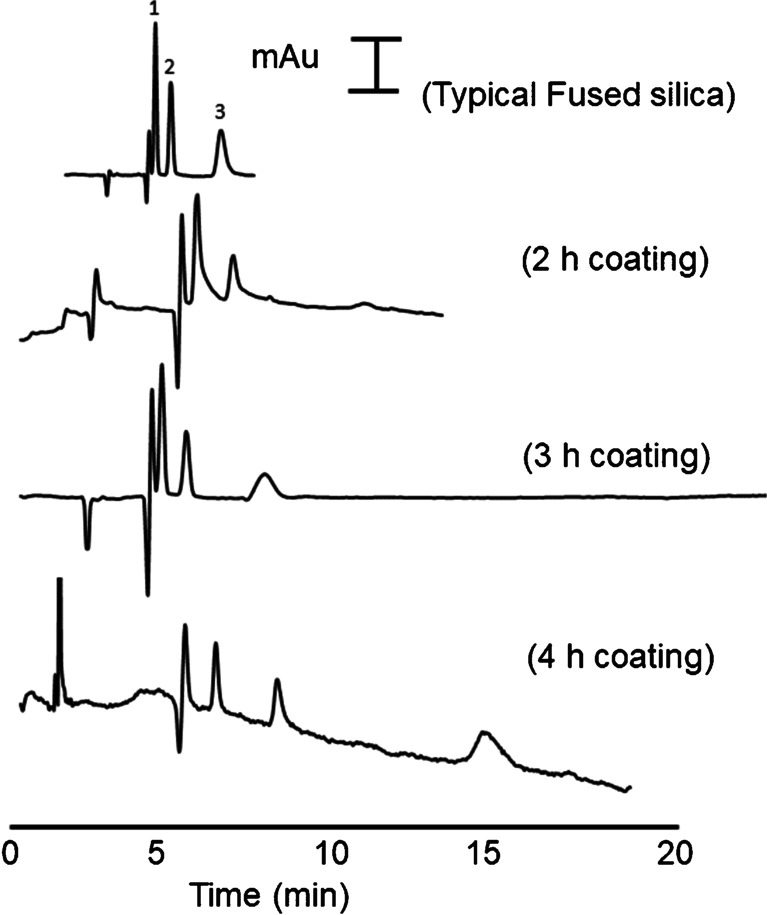
Effect of coating time on the OT-LC separation of phenones (1, butyrophenone; 2, valerophenone; and 3, hexanophenone) using SNSs-coated capillaries (mobile phase: 0.25 mM CTAB in 25 mM borate buffer, pressure: 50 mbar, injection: 5 s at 25 mbar, and detection wavelength: 200 nm)

Therefore, the concentration of ammonia solution and the ratio of ammonia/water/ethanol/TEOS were studied through a one-at-a-time procedure and the conditions providing the best results were obtained as 6.5 mL TEOS, 21 mL ethanol, 2 mL MQW, and 5.6 mL ammonia solution (0.5 M). Additionally, surfactant modifiers with different concentrations were dissolved in the MQW portion (2 mL) and added to the sol–gel environment (which will be discussed in later sections). The coating time showed a great influence on the success of the coating properties, including thickness and performance of chromatographic separation. Coating times in the range of 1–6 h were examined. Coating times less than 2 h did not provide enough thickness to achieve better separation compared to the conventional capillaries, while > 4 h resulted in column blockage and deterioration of the peak shapes due to the over formation of SNS inside the capillary. The highest retention for the OT-LC analysis of phenones was achieved after 3 h of dynamic coating, indicating the formation of a high surface area stationary phase. Therefore, 3 h was selected as the optimum coating time. The chromatograms and the changing trend of retention factors (*k′*) of phenones, against coating time, separated by OT-LC are presented in Fig. 2.

Fused silica capillaries with different internal diameters (i.e., 25, 50, 75, and 150 µm) were tested. The results showed that < 50 µm i.d. capillaries were very prone to clogging while > 75 µm i.d. needed long times to be coated. So, 50 µm capillary was selected for further studies. Post-coat drying time and temperature were also investigated. Accordingly, overnight heat drying in an oven (at 95 °C) was found to be the best strategy. The drying process caused a decrease in the gel volume (which was investigated by the retention factor of phenones) due to the evaporation of liquid through the silica pores [41].

Attempts were made to use a digital stereomicroscope as a tool for quick pre-evaluation of the prepared SNS-coated capillaries, but it could barely distinguish a clear difference between an SNS-coated and a regular capillary, particularly when the coated layer was thin. Therefore, the surface morphology of the inner walls of the coated capillaries was assessed using cross-sectional SEM micrographs. Furthermore, the performance of the prepared capillaries was evaluated by the separation of three neutral analytes (butyrophenone, valerophenone, and hexanophenone). The BGS/mobile phase was 25 mM borate buffer in 0.2 mM CTAB, and the analytes were injected hydrodynamically at 25 mbar for 5 s. The CTAB micellar solution created a bilayer (admicelles) at the interface of the solid/liquid interface (within 50–200 μm), acting as a soft stationary pseudo-phase for the chromatographic separations of charged and neutral species, demonstrated in our previous studies [46]. OT-CEC and OT-LC separations were carried out at − 10 kV and 50 mbar, respectively. The retention factor (*k′*) was calculated using *k* = (*t*_r_—*t*_0_) /* t*_0_, in which “*t*_0_” is void time and “*t*_r_” is retention time.

One of the most common routes to obtaining mesoporous silica nanoparticles is the use of a templating agent, especially surfactants that act as structure-controlling agents [47]. This study aimed to achieve minimal nanoparticle aggregation by adding different modifiers including cationic (CTAB), anionic (SDS), and neutral (PVP) surfactants to the optimized synthesis recipe. For this purpose, different concentrations (0.25, 0.5, and 2%, w/v) of the surfactants in 2 mL of MQW were added to the synthesis media. Except for PVP, concentrations over 0.25% of the modifiers caused a blockage in the column. Additionally, post-coating overnight drying led to blockage of the SNS-coated columns with SDS and CTAB modifiers, even at a concentration of 0.25%. The prepared columns were evaluated in OT-LC separation of phenones. As can be seen from the results summarized in Table 1, the SNS-coated column, modified with a 2% PVP modifier, provided the highest retention factor, about twice the value obtained for SDS and CTAB.

**Table 1:** Effect of the modifier percent and drying procedure on the separation performance of SNSs-coated columns for OT-LC separation of phenones (the *k′* values are for hexanophenone)

Column coated with	Modifier type	Modifier percent	Post-coating immediate drying (3 h, 95 °C)	Post-coating overnight drying (95 °C)	*k′* (hexanophenone)
SNS	–	–	×	✓	0.85
SNS-SDS	SDS	0.25%	‏‏✓	×	0.85
	SDS	0.25%	×	✓	Blocked
SNS-CTAB	CTAB	0.25%	✓	×	0.810
	CTAB	0.25%	×	✓	Blocked
SNS-PVP	PVP	2%	✓	×	0.833
	PVP	2%	×	✓	1.608

**Fig. 3:**
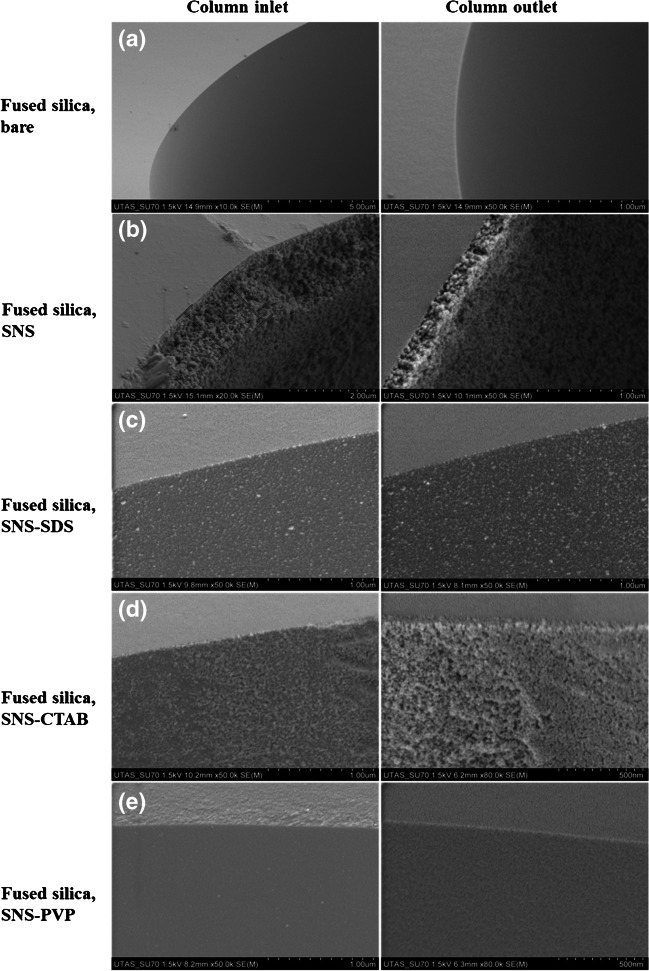
SEM micrographs of inner wall of an uncoated fused silica capillary (**a**), and capillaries coated with SNS (**b**), SNS and SDS modifer (**c**), SNS and CTAB modifer (**d**), and SNS and PVP modifer (**e**)

The SNS-coated columns (34 cm long, 50 µm i.d.) were further evaluated by recording their cross-sectional SEM micrographs (Fig. 3). As is clear from the SEM images, the addition of surfactants, regardless of their nature, caused an extreme decrease in the size of the SNS. However, they did not make any improvement in the chromatographic separation, as was shown in Table 1. As depicted in Fig. 3, SEM micrographs show that the maximum surface porosity is created by the formation of SNS using TEOS alone, without the need for modifiers, proving the higher retention times for the phenones.

The SNS-coated capillaries were also modified with APTES and applied for the OT-LC (pressure-driven) and OT-CEC (voltage-driven) separation of phenones. As represented in Supplementary Material, Fig. S2, the obtained results were not satisfactory. It showed a poor separation for the phenones in the first run and worsened in subsequent consecutive runs, especially for the OT-CEC analysis, possibly due to the breaking of the bond between the APTES and silica and the leaching of APTES from the column.

### Modification of the SNS-coated columns with octadecyl group

**Fig. 4:**
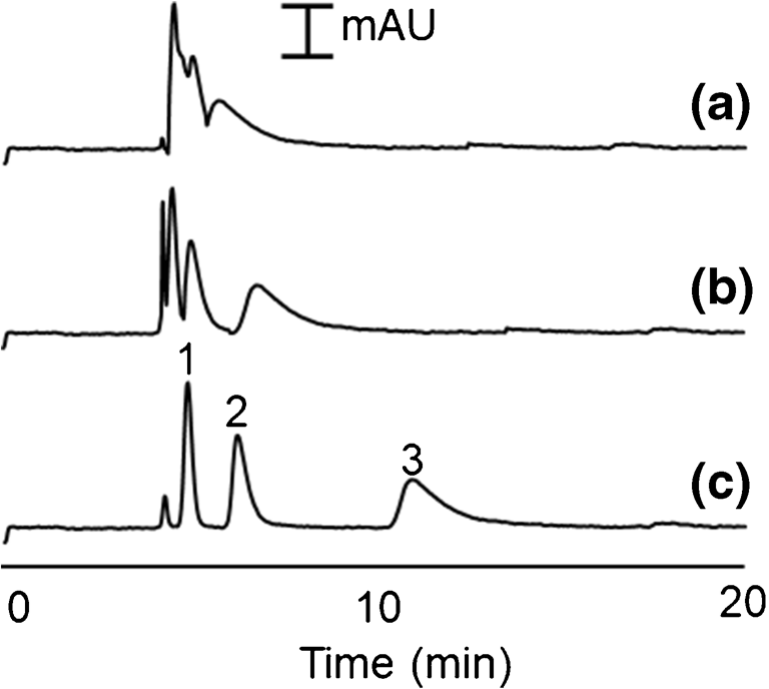
Representative chromatograms for the OT-LC separation of neutral phenones on SNS-OTS modified columns, prepared using different solvents, a pure ethanol, b mixture of toluene/ethanol (4/1), and c pure toluene. The sample of phenones (1, butyrophenone; 2, valerophenone; and 3, hexanophenone) was prepared in the mobile phase (mobile phase: 25 mM phosphate buffer with pH 7.0, pressure: 50 mbar, injection: 5 s at 25 mbar, and detection wavelength: 200 nm)

The surface of the SNS coated on the inner walls of the fused silica capillary was further functionalized with OTS to prepare a reverse-phase stationary phase for OT-LC separation. It was found that the functionalization of the surface of the SNS particles (already coated on the inner walls of the capillary) is done effectively. But with the formation of OTS, the UV-detection window of the column becomes blurred, and by preventing the passage of light, it causes a sharp decrease in sensitivity. Therefore, OTS was gradually added to the reaction medium. To achieve the highest separation performance, the type of solvent for the addition of TOS was also studied. Accordingly, ethanol and toluene were evaluated as solvents to coat fused silica capillaries with SNS, functionalized with OTS (1%). According to the work done by Grunze et al., functionalization of fused silica using OTS in ethanol solvent can lead to incomplete surface capping due to the reaction between ethanol and the silanol groups on the surface of silica [48]. This reaction prevents the bonding of OTS with silanol groups and creates an imperfect functionalized surface, as is clear from the results obtained for the OT-LC separation of phenones, depicted in Fig. 4. Hereupon, pure toluene and a mixture of toluene/ethanol (4/1) were applied for the functionalization of the SNS-coated columns with OTS. As is clear from Fig. 4c, pure toluene provided better resolution for the pressure-driven separation of phenones.

It has been amply demonstrated that molecular orientation in methyl-terminated organosilane coatings on silica surface is dependent on the alkyl chain length. Long-chain organosilanes create well-ordered monolayers while oppositely, short-chain silanes lead to disordered multilayer films. Indeed, long-chain organosilane-like OTS molecules aggregate in an island-type growth behavior during film formation. Smaller secondary nucleation islands of OTS grow between the primary areas until the film is complete [49]. The size and shape of the aggregates (islands) and the rate of nucleation depend on the preparation conditions. Amino­terminated silanes have been demonstrated to be disordered, possibly because of chemical interactions of the amino groups with the substrate [50]. Accordingly, it was thought that if a mixture of OTS (as a source of long-chain alkyl) and APTES (as a source of amino-terminated short-chain alkyl) is used, a combination of ordered and disordered layers may provide better adsorption and separation properties. For this purpose, a 1:1 mixture of 1% OTS and 1% APTES solutions in toluene was used for the functionalization of the SNS-coated columns. As mentioned earlier, APTES functionalized SNS-coated columns were not stable, and during chromatographic separations, the APTES functional group was flushed out of the column due to the instability of their chemical bonds with silica. Figure S3 shows the chromatograms resulting from the separation of phenones using SNS-coated capillaries functionalized with OTS and OTS in the presence of APTES. OTS alone (Fig. S3a) provides better separation probably because of the more organized bonding of OTS groups on the surface of SNS, in comparison with the less organized stationary phase containing OTS and APTES (Fig. S3b). On the other hand, the presence of APTES creates a more hydrophilic stationary phase, which can be considered as another potential reason for the shorter retention times of phenones using the SNS-coated surface functionalized with OTS and APTES.

Additionally, SEM micrographs of an SNS-coated capillary functionalized with OTS/APTES (SNS-OTS/APTES), a typical fused silica capillary functionalized with OTS/APTES (FS-OTS/APTES), and a typical fused silica capillary functionalized with OTS (FS-OTS) were recorded (Fig. S4). Even though the separation performance of the SNS-OTS/APTES coated column is not better than that of the SNS-OTS coated column, SNS-OTS/APTES provides a larger surface area, as can be seen in Fig. S4a.

### EOF evaluation for the SNS-OTS-modified capillary columns

**Fig. 5:**
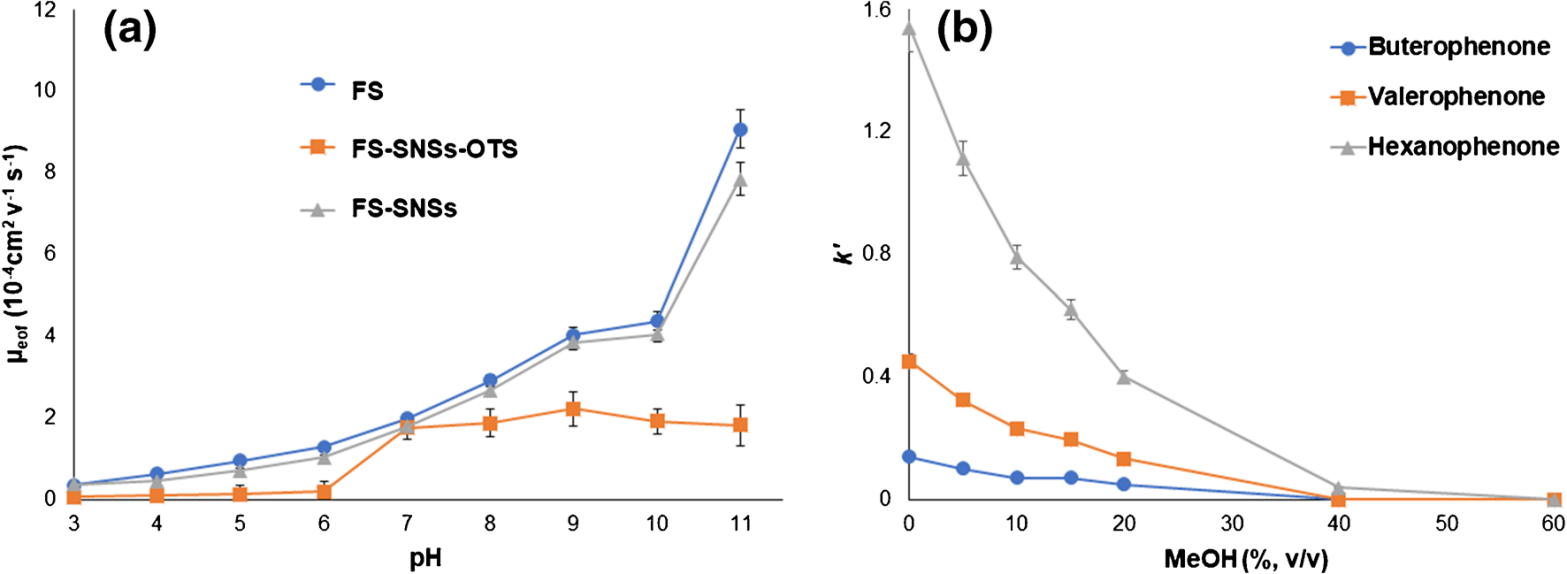
** a** The effect of BGS’s pH on the EOF mobility of a typical fused silica capillary, FS, a SNS-coated fused silica capillary, FS-SNS, and a SNS-OTS-coated fused silica capillary, FS-SNS-OTS (BGS: for pH 3–5 was 30 mM sodium citrate/citric acid buffer, for pH 6–8 was 20 mM PBS, for pH 9–10 was 25 mM borate buffer, and for pH 11 was 25 mM sodium hydrogen orthophosphate/sodium hydroxide, applied voltage: + 20 kV; injection, 5 s at 25 mbar, detection wavelength: 200 nm, EOF marker: 1% v/v acetone; column: 31 cm capillary with 22.5 cm effective length, temperature: 25 ℃), and **b** correlation between the retention factor and methanol percentage in the mobile phase (25 mM borate buffer at pH 9.5) for the OT-LC separation of butyrophenone, valerophenone, and hexanophenone.

An essential matter of great importance in OT-CEC is EOF stability, arising from a stable superficial modification [51]. Herein, EOF was calculated according to the following formula [52]:

$${\mu }_{eof}={L}_{t}.{L}_{eff}$$

where *L*_*t*_ is the total length of the capillary (34 cm), *L*_*eff*_ is the effective length of the capillary (25.5 cm), *V* is the applied voltage (+ 20 kV), and *t*_*0*_ is the migration time of a neutral marker (1% acetone in the BGS). The EOF was evaluated by gradually increasing BGS pH over the range of 5.5–10.5, which steadily increased the ionization of the silanol groups on the column surface. Figure 5a demonstrates a comparison between the EOF mobility of the neutral marker on a typically fused silica capillary (FS), an SNS-coated fused silica capillary (FS-SNS), and an SNS-OTS coated fused silica capillary (FS-SNS-OTS). As is clear from the results, the SNS-OTS-modified capillary exhibited a constant EOF, significantly lower than the bare fused silica, at pHs above 7.5. This fact demonstrates an effective modification of the SNS surface by stable layers of C_18_ groups.

### Retention mechanism and reproducibility

To study the retention mechanism, the retention factor (*k'*) was correlated to the percentage of organic solvent in the mobile phase for the OT-LC separation of the phenones (butyrophenone, valerophenone, and hexanophenone). As depicted in Fig. 5b, the retention factors decrease with increasing methanol percentage, indicating the dominance of a reverse-phase mechanism, caused by the hydrophobic interaction of the C_18_ hydrophobic tail with the analytes [53].

The reproducibility of the retention time was investigated for the separation of the neutral analytes. The results are summarized in Table 2. The results showed that retention time’s RSDs were 1.17*–*1.35% for intra-day and 2.59*–*3.08% for inter-day analyses, which indicates the stability of the SNS-C_18_ stationary phase and the high degree of reproducibility of the functionalization process. Column-to-column RSDs over the range of 3.58*–*4.92 indicates acceptable reproducibility for the coating/functionalization process.

**Table 2:** Intra-day, inter-day, and column-to-column reproducibility obtained by the OT-LC analysis of the neutral analytes using SNS-OTS functionalized fused silica capillary columns

Analyte	RSD of retention time (%, *n* = 3)		
	Intra-day	Inter-day	Column-to-column
butyrophenone	1.17	2.59	3.58
valerophenone	1.25	2.68	4.46
hexanophenone	1.35	3.08	4.92

### Functionalization of the SNS-coated fused silica capillaries with *β*-CD

Synthesis of a stable silica-based stationary phase with a surface-bound chiral group had been of great interest for chromatographic separations of chiral compounds, particularly in drug development. Chiral molecules play a prominent role in the preparation of medicines. In this regard, many research studies have been carried out for the preparation of OT-LC and OT-CEC columns, using *β*-CD and its derivatives and composites [54–56]. In this research, to prepare a permanent chiral stationary phase, the possibility of two methods “post-functionalization of SNS with *β*-CD” and “adding *β*-CD during the in situ formation of SNS” on the inner surface of fused silica capillaries was studied. Different synthesis recipes were tested to optimize the synthesis procedure to fit the purpose. The results showed that post-functionalization of SNS-coated capillaries was significantly more effective.

The *β*-CD functionalized column was applied for the OT-CEC separation of dichlorprop racemic mixture and satisfactory results were obtained, as shown in Fig. S5. A potassium dihydrogen phosphate buffer (20 mM, pH 7.0) was used as the BGS at − 15 kV. The chiral stationary phase was stable for six consecutive runs. Though the prepared chiral stationary phase provided acceptable enantioselectivity, it did not show long stability and after five consecutive analyses, the peaks of the enantiomers almost overlapped. The low stability of chiral stationary phases is a general problem, and most laboratory-made chiral stationary phases withstand approximately less than 100 analyses [14, 57]. This is probably because of the instability of *β*-CD bonded to the surface of SNS, caused by the high hydrophilicity of *β*-CD and its tendency to dissolve in the BGS [58]. To address this limitation, different derivatized cyclodextrins such as Heptakis-*β*-CD and quaternary ammonium alpha-CD were tested, but no notable improvement in stability was observed.

### Application of SNS-C_18_-modified column to real samples

To evaluate the reliability and applicability of the developed SNS-TOS-modified columns, they were applied for the OT-LC and OT-CEC analysis of real samples with complex matrices including clove whole extract and BSA digest. Two alkenylbenzenes (eugenol and methyl eugenol) were analyzed using OT-LC in a clove whole extract. The samples were prepared as mentioned in “Sample preparation.” Different conditions were examined, and a 100 mM phosphate buffer solution (pH 5.8) and 50 mbar were selected as the optimal mobile phase and separation pressure. The samples were diluted using the mobile phase to achieve suitable concentrations. The sample chromatograms depicted in Fig. S6, indicate that the SNS-TOS-modified column has successfully retained and separated the analytes in the complex matrix of the herbal extract.

**Fig. 6:**
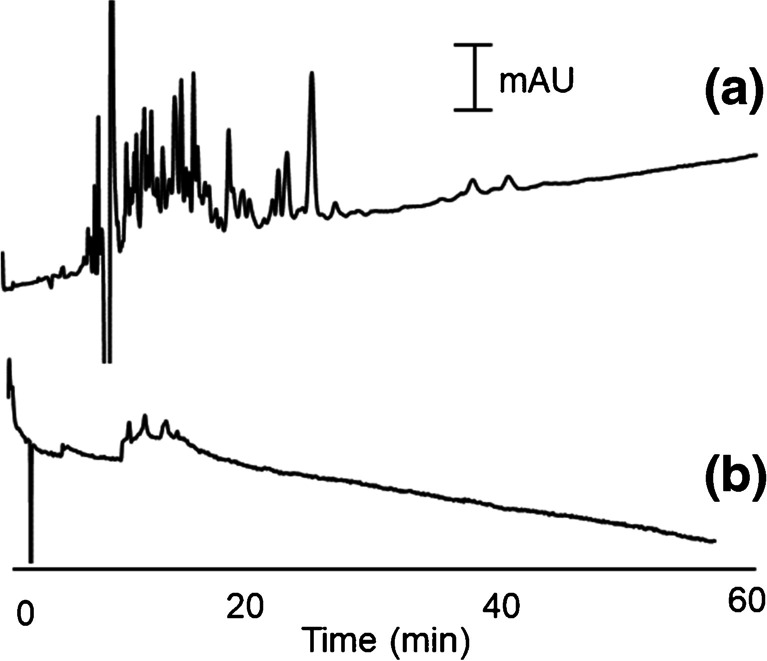
Representative electropherograms for OT-CEC analysis of BSA digest using capillary columns coated by (**a**) SNS-OTS and (**b**) PDDA-PSS (BGS: 500 mM FA in 20% ACN, applied voltage: + 10 kV, injection: 10 s at 25 mbar, and detection wavelength: 200 nm)

The functionalized column was also applied to evaluate the tryptic digest of BSA, containing a mixture of numerous peptides in a complex matrix. To have a basis for comparison of the separation performance, a normal silica capillary was modified with poly(diallyldimethylammonium) chloride/polystyrene sulfonate (PDDA-PSS) [59] and tested under the same condition. PSS would create a negatively charged surface as the partial negative charged surface resulted from the unbonded silanol groups in an SNS-OTS-coated capillary. PDDA provides a positively charged surface for the immobilization of PSS and also prevents proteins from binding to the capillary surface. As represented in Fig. 6, the resolving power and peak capacity obtained on the SNS-TOS-modified capillary column is significantly higher than the PDDA-PSS column. Therefore, the developed column can be considered a new prospect for peptide separation.

The SNS-OTS-functionalized column was also applied for the OT-LC and OT-CEC separation of three NSAIDs in their ionized and molecular forms, respectively. The p*k*_*a*_ of ketoprofen, ibuprofen, and fenbufen are 5.9, 5.2, and 4.2, respectively. Therefore, for the OT-LC analysis, mobile phase pH was chosen to be near the p*k*_*a*_ values to maintain the analytes at molecular forms. As can be deduced from the results, the OT-CEC (Fig. S7a) separation showed a higher selectivity than OT-LC (Fig. S7b).

## Conclusion

The era of bulky chromatography devices with high solvent consumption is over and the new world requires miniaturized, portable, and field-applicable devices. Regarding liquid chromatography, the most important necessity to fulfill this objective is the development of high-resolving power capillary columns. Herein, to increase the resolving power of the regular silica capillaries, they were first coated with silica nanospheres (SNS) to increase the sorption ability and effective surface area. Then, the surface of SNS was functionalized by C_18_, amino, and *β*-CD groups. The prepared columns were then evaluated for the OT-LC and OT-CEC separation of neutral, charged, and chiral species, and the results showed promising aspects in terms of resolving the power and stability of the columns. The most critical need is to improve the in situ dynamic method to create a more uniform SNS layer at the inlet and outlet of the column, without blocking during the synthesis process. In this case, smaller diameter columns can also be easily coated through this method, paving the way for the development of miniaturized LC systems. Therefore, to increase the stability and chromatographic performance of the produced columns, more detailed studies should be carried out on this coating technique.

## Supplementary information

Below is the link to the electronic supplementary material.

Supplementary file1 (PDF 558 KB)

## Declarations

### Source of biological material

N/A. No research was performed involving biological material.

### Statement on animal welfare

N/A. No research was performed involving animals.
