# Supplementary material for: Dynamic in situ growth of bonded-phase silica nanospheres on silica capillary inner walls for open-tubular liquid chromatography
Source: Anal Bioanal Chem. 2023 Jun 23;415(20):4923–34. doi: 10.1007/s00216-023-04798-1 (PMC10386930; doi:10.1007/s00216-023-04798-1)
Supplement: Supplementary file 1 — Supplementary file1 (PDF 558 KB) [file 216_2023_4798_MOESM1_ESM.pdf]

Supplementary Information (SI)

Dynamic in-situ growth of bonded-phase silica nanospheres on silica capillary inner walls for open-tubular liquid chromatography

Mohamed Adel Ahmed [a], Alireza Ghiasvand [a,b,*] Joselito P. Quirino [a,**]

[a] *Australian Centre for Research on Separation Science (ACROSS), School of Natural Sciences, University of Tasmania, Hobart, TAS 7001, Australia*

[b] *Department of Chemistry, Lorestan University, Khoramabad, Iran*

*** Corresponding Author:**

Alireza Ghiasvand

E-mail: alireza.ghiasvand@utas.edu.au

ORCID ID: https://orcid.org/0000-0002-4570-7988

**** Corresponding Author:**

Joselito P. Quirino

E-mail: joselito.quirino@utas.edu.au

ORCID ID: https://orcid.org/0000-0002-8275-9672

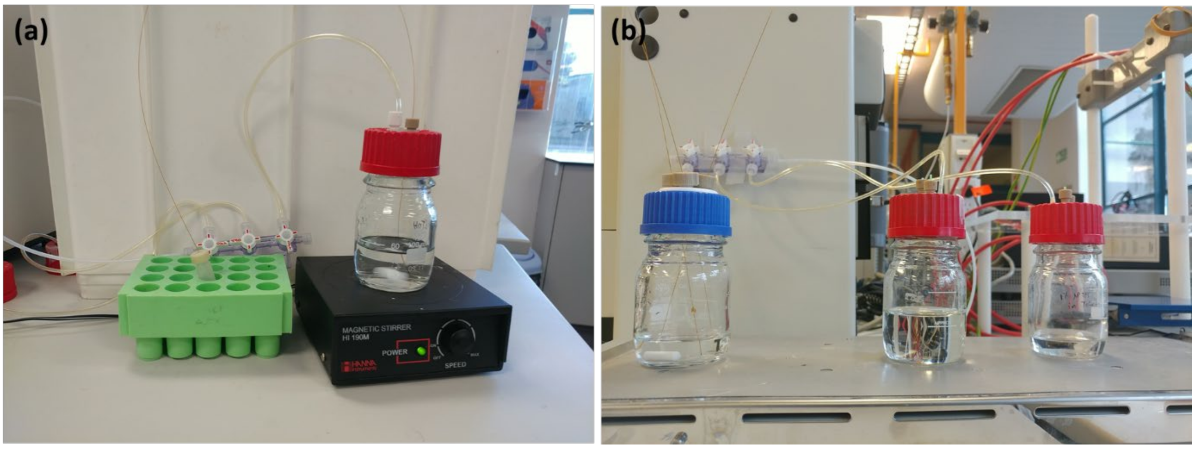

Fig. S1. (a) A pressurized chamber suitable for coating one column at a time and (b) three series connected coating chambers for coating of multiple columns with (different coating conditions) at a time.

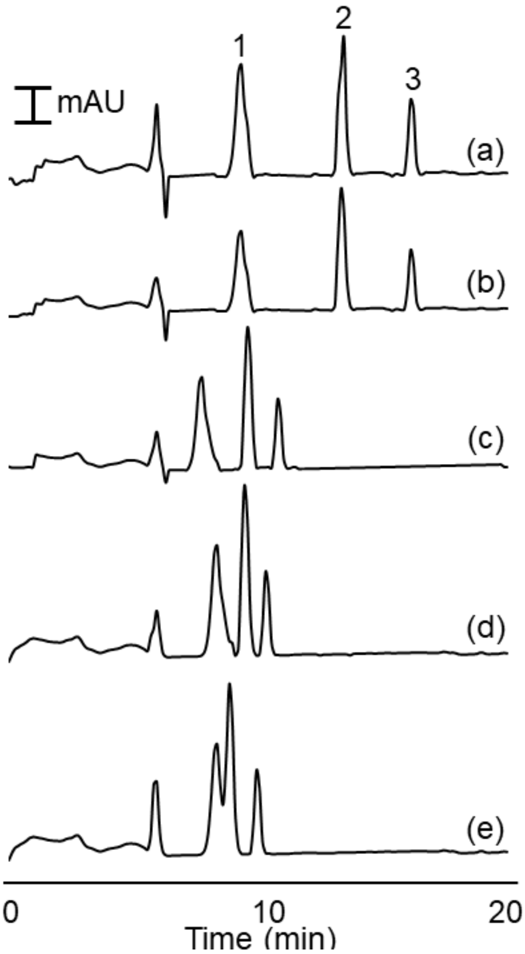

Fig. S2. Representative chromatograms for 5 consecutive runs (a-e) obtained from pressure OT-LC separation of neutral analytes using SNS-APTES coated capillary. The sample solution of neutral phenones (1. butyrophenone, 2. Valerophenone, and 3. Hexanophenone) were

prepared in the mobile phase (mobile phase: 0.5 mM SDS in 25 mM borate buffer, separation pressure: 50 mbar, injection: 5 s at 25 mbar, detection wavelength: 200 nm).

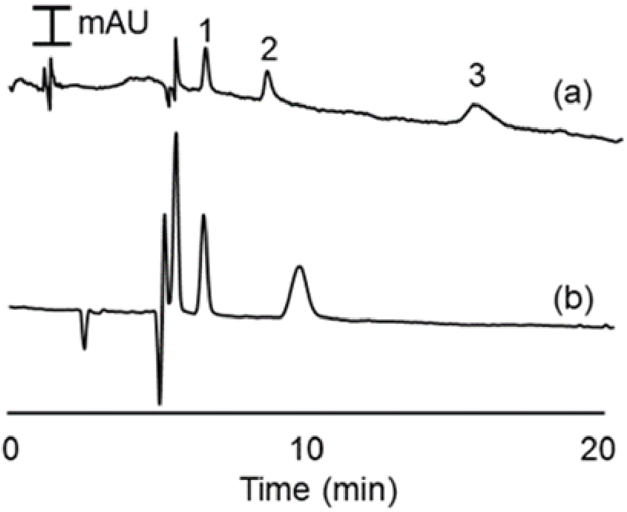

Fig. S3. Representative chromatograms of phenones (1. butyrophenone, 2. valerophenone, and 3. hexanophenone) separated using SNS-coated columns functionalized with OTS (a) and OTS in the presence of APTES (b). (Mobile phase: 25 mM phosphate buffer pH 7.0, pressure: 50 mbar, injection, 5 s at 25 mbar, detection wavelength: 200 nm).

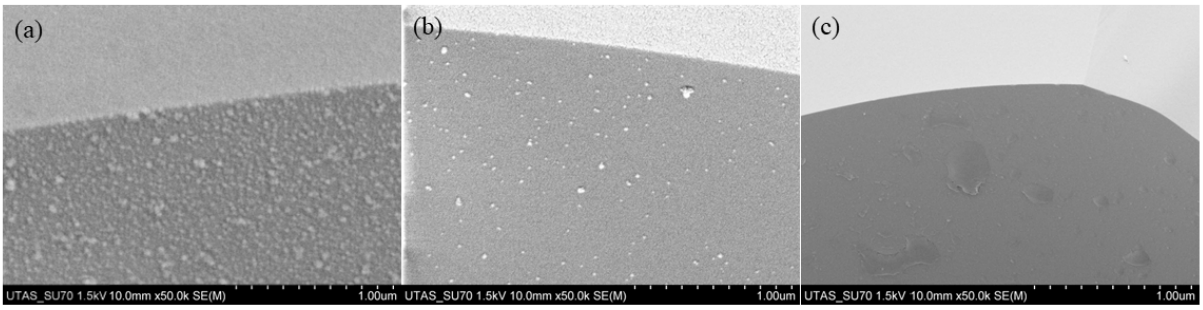

Fig. S4. SEM micrographs of (a) a SNS-coated capillary functionalized with OTS/APTES, (b) a typical fused silica capillary functionalized with OTS/APTES, and (c) a typical fused silica capillary functionalized with OTS.

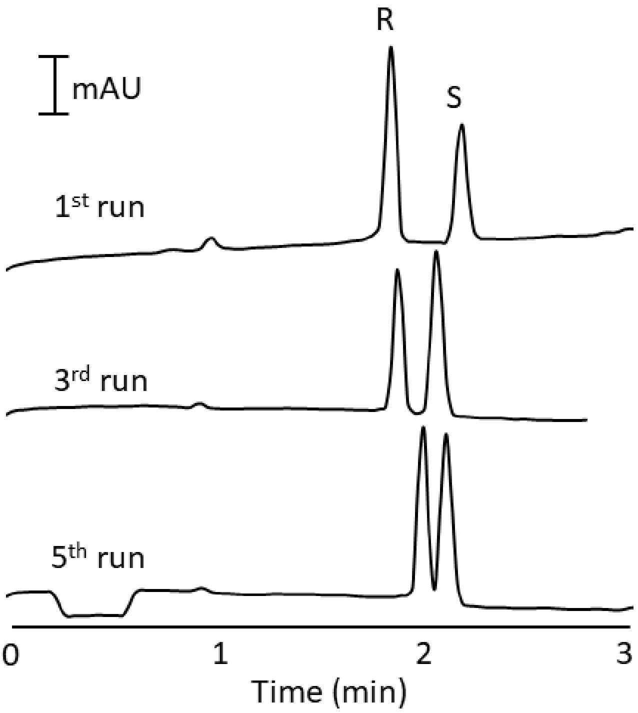

Fig. S5. Representative electropherogram for the OT-CEC separation of a 50 μg mL^{-1} racemic dichlorprop solution using a fused silica capillary column coated with SNS-β-CD. BGS was 20 mM KH_2PO_4 (pH 7.0) at -15 kV.

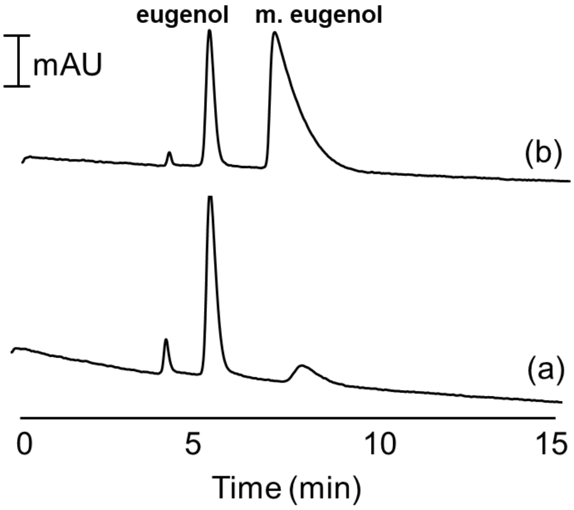

Fig. S6. OT-LC analysis of alkenylbenzenes in (a) a clove whole extract sample and (b) the whole extract sample spiked with 25 μg mL^{-1} of the analytes using a fused silica capillary coated by SNS-OTS (mobile phase: 100 mM phosphate buffer at pH 5.8, pressure: 50 mbar, injection: 5 s at 25 mbar, detection wavelength: 200 nm).

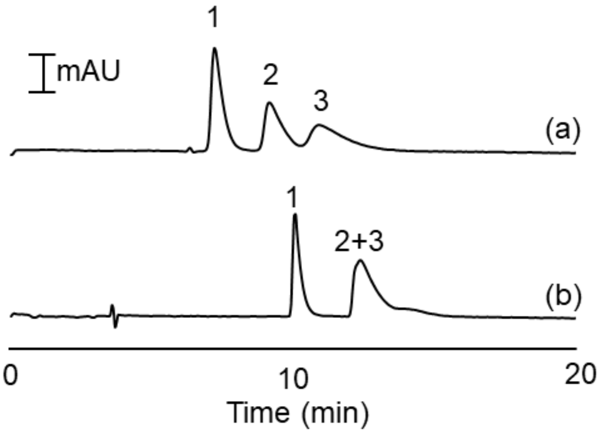

Fig. S7. Representative chromatogram/electropherogram for the OT-CE (a) and OT-LC (b) separation of ketoprofen (1), ibuprofen (2), and fenbufen (3) using SNS-OTS functionalized capillary columns (mobile phase/BGS: 10 mM ammonium acetate at pH 5.5, injection: 5 sec at 25 mbar, and pressure/voltage: 50 mbar/+10 kV).
